# Poloxamer-Based Hydrogel as Drug Delivery System: How Polymeric Excipients Influence the Chemical-Physical Properties

**DOI:** 10.3390/polym14173624

**Published:** 2022-09-01

**Authors:** Elisa Brambilla, Silvia Locarno, Salvatore Gallo, Francesco Orsini, Carolina Pini, Marco Farronato, Douglas Vieira Thomaz, Cristina Lenardi, Marco Piazzoni, Gianluca Tartaglia

**Affiliations:** 1 Department of Pharmaceutical Sciences, Section of General and Organic Chemistry Section “A. Marchesini”, University of Milan, 20133 Milan, Italy; 2 Department of Physics “Aldo Pontremoli”, University of Milan, 20133 Milan, Italy; 3 Department of Biomedical, Surgical and Dental Sciences, University of Milan, 20100 Milan, Italy; 4 National Enterprise for NanoScience and NanoTechnology (NEST), Istituto Nanoscienze-CNR and Scuola Normale Superiore, Piazza San Silvestro 12, 56127 Pisa, Italy

**Keywords:** poloxamer 407, thermogelling hydrogel, hydrophilic polymers, drug delivery system, chlorhexidine digluconate, doxycycline hyclate

## Abstract

Thermogelling amphiphilic block copolymers have been widely investigated in the development of pharmaceutical drug carriers. In particular, thermosensitive gels based on poloxamer 407 (P407) have great potential for periodontal disease treatment, thanks to their ability to be liquid at room temperature and become viscous gels at body temperature. However, some problems, related to short in situ residence time, reduce their feasible clinical use. Thus, in order to improve the effective applicability of these materials, we studied how P407 thermogels are affected by the pH and by the inclusion of different hydrophilic polymers, used as excipients for increasing the gel stiffness. For this scope, a complete chemical-physical characterization of the synthesized gels is provided, in terms of determination of sol-gel transition temperature, viscosity and erosion degree. The data are correlated according to a statistical multivariate approach based on Principal Component Analysis and their mucoadhesion properties are also tested by Tapping mode-Atomic Force Microscopy (TM-AFM) imaging. Finally, we studied how the different P407 formulations are able to influence the release pathway of two antibacterial drugs (i.e., chlorhexidine digluconate and doxycycline hyclate) largely used in oral diseases.

## 1. Introduction

In recent years, research about self-assembled systems has gained increasing attention as they have demonstrated potential application in drug delivery due to their many advantages including improving solubility of drugs, biocompatibility, biodegradability, high efficiency for drug loading, reducing drug toxicity, controlling drug release rate and protection against biochemical degradation [1]. Several types of self-assembled structures have been studied to develop new drug carriers involving micellar, hydrogels, vesicles, polymers, microcapsules, liposomes, cubosomes, colloidosomes, etc. [2,3,4]. Among different biomaterials that can be used to fabricate smart systems, stimuli-sensitive polymers have been gained a great attention due to their unique properties that find applications in several fields of biomedical sciences. For example, drug delivery systems and tissue engineering applications take great advantages of pH- and photo-responsive materials with temperature sensitive properties [5,6,7]. An interesting class of biomaterials employed in different type of drug delivery systems is that of hydrogels. These materials are a unique class of three dimensional cross-linked polymeric networks of physically or/and chemically cross-linked polymers which can imbibe and retain a large amount of aqueous solvents and biological fluids in intermolecular space [8,9]. They possess also a degree of flexibility very similar to natural tissue due to their large water content. The ability of hydrogels to absorb water arises from hydrophilic functional groups attached to the polymeric backbone, while their resistance to dissolution arises from cross-links between network chains. The generation of cross-linked structure can be promoted by a variety of stimuli, such as variation of temperature, pH or irradiation [10,11].

Among this class, thermogelling amphiphilic block copolymers have been widely investigated in the development of pharmaceutical drug carriers, thanks to their capability to self-assemble into micelles. The micelles formation is a consequence of the different solubility of the polymer units that undergo to aggregation after reaching the Critical Micelle Concentration (CMC), by exploiting physical or chemical interactions between the units in order to find the lowest-energy configuration. Since the solvation of the amphiphilic block copolymers is strongly dependent on temperature, another important parameter to take into account for self-assembling is the Critical Micellization Temperature (CMT), below which the polymer exists as unimer, and above which, unimers and aggregates coexist [12]. These amphiphilic block copolymers at a given concentration are characterized by specific CMT.

Poloxamers, available also under the trademark Pluronics^®^, belong to this class of material. They are water-soluble nonionic A-B-A and B-A-B triblock copolymers, where A is poly(ethylene oxide) (PEO) and B is poly(propylene oxide) (PPO). The PEO blocks and the PPO block constitute the hydrophilic and hydrophobic portions, respectively [13]. Depending on blocks length, the core can assemble into various supramolecular structures characterized by different and versatile morphologies. One of the most interesting features of poloxamers is the possibility to modulate the CMT below body temperature, by increasing the concentration of the polymer. Moreover, the thermogelling process is reversible, that means poloxamers are liquid at room temperature, but they assume a gel form when administrated at body temperature, which makes them attractive candidates for the development of in situ gel depot, prolonging the pharmacokinetics of the drug dispersed inside [14]. Poloxamer 407 (P407), which is also known as Pluronic^®^ F127, is one of the most studied members of poloxamer family thanks to its good solubilizing capacity, low toxicity, good drug-release characteristics, and its compatibility with numerous biomolecules and chemical excipients [15,16,17]. Recently, the applicability of P407 in the field of periodontal diseases was intensive investigated and several in situ gel formulations for local drug delivery have been proposed [18,19,20]. There are many advantages in using such formulations respect to systems currently available on the market, especially in terms of adaptability to the pocket geometrical structure and its non-invasive applicability into the target situ. However, some problems related to short in situ residence time, reduce the clinical use of P407 in periodontal treatments. The incorporation of other biocompatible polymers as excipients in the P407 formulation could potentially solve these issues by increasing the strength and the mucoadhesion (which is defined as attractive force at the interface between a pharmaceutical formulation and mucus or mucous membrane), ensuring a sustained release of the dispersed drugs.

However, it is of utmost importance to predict gelation behavior after the addition of excipients into a copolymer formulation. In fact, the interactions between the block copolymer and added molecules can alter the CMC, CMT and other physicochemical properties. The change in solution properties is a useful indicator for the strength of such interactions. Therefore, the aim of this work is the optimization of P407 formulation in order to serve specific needs such as injectable liquid form at room temperature, mucoadhesive gel form below 30 °C, pH close to neutrality and biocompatibility. Next, the ability of P407 formulations to release active molecules was investigated. For this scope, two hydrophilic molecules widely used in periodontal diseases was selected. The first one is chlorhexidine digluconate (CHX) which is a bis-biguanide with a broad spectrum of activity that includes antibacterial action on gram-positive and gram-negative [21]. The other one is Doxycycline hyclate (DOX) is a bacteriostatic agent belonging to the tetracyclines family [22]. Finally, this work gives in depth investigation about physicochemical properties, such as determination of transition temperature, viscosity and erosion degree of P407 formulations in presence of different additives (Xanthan Gum; Carrageenan; hydroxypropyl methylcellulose, and polyvinylpyrrolidone) in order to connect them to their utility in formulating delivery systems and in particular the influences of these properties on the drug release pathway.

## 2. Materials and Methods

### 2.1. Materials

P407 (Pluronic F-127), carrageenan (E407), xanthan gum (XG), (hydroxypropyl)methyl cellulose (HPMC) and polyvinylpyrrolidone (PVP) were purchased from Sigma-Aldrich (St. Louis, MO, USA), doxycycline hyclate (DOX), chlorhexidine digluconate (CHX) were purchased from Thermo Fisher Scientific (Waltham, MA, USA). All batches of hydrogel were prepared using analytical grade reagents. All batches of hydrogels were prepared using ultrapure water (resistivity 18.2 MΩ·cm) obtained by a water purification system (Milli-Q^®^ Direct, EMD Millipore, Darmstadt, Germany).

### 2.2. Preparation of the Gels

The gel formulations were prepared by the modification of “cold method” reported in literature [23]. The thermogelling systems only composed by P407 were prepared by adding the correct amount of the polymer (13, 15, 18, 20% *w/w*) in cold ultrapure water or PBS 1× and the mixture was stirred at 4 °C overnight. The thermogelling systems presenting an additive were prepared by dispersion of HPMC (0.5 or 1% *w/w*), PVP (4% *w/w*) or XG (0.2% *w/w*) at room temperature or E407 (0.2% *w/w*) at 70 °C in ultrapure water or in PBS 1× solution. After complete dissolution of the additives, the solution was cooled to 4 °C and the amount of P407 was added. The preparation was stirred at 4 °C for 24 h before analysis to ensure the complete polymer wetting (see Table 1 and Table 2 report the final composition of all tested preparation). Finally, the correct amount of the active ingredient (CHX 0.2% *w/w*; DOX 3% *w/w*) was added and the preparation was stirred for 1 h at 4 °C.

### 2.3. pH Evaluation

pH was evaluated by measuring the solution at room temperature with a pH meter multi-thermometer VWR (Radnor, PA, USA), Traceable calibration control company.

### 2.4. Measurement of Solution-Gel Transition Temperature (T_sol/gel_)

Method A. The determination of T_sol/gel_ was the modification of method described by Patlolla [24]. The formulation (3 mL) was placed in a test tube and immersed in water bath attached to a thermostat at 10 °C. The gelation was evaluated at every 1 °C increments by tilting the test tubes and gelation was considered when meniscus no longer moved.

Method B. The second method was the adjustment of the reported Choi’s procedure [25]. A 5 mL transparent vial containing a magnetic bar and 3 mL of the preparation gel was placed in a water ice bath at 4 °C. A digital thermosensor, with a sensitivity of 0.1 °C (m) connected to a thermistor was immersed in the gel. The gel was heated at the rate of 1 °C/min with continuous constant stirring. When the magnetic bar stopped moving due to the gelation, the measured temperature was considered as a gelation temperature.

The reported T_sol/gel_ were obtained as average between the results obtained from the two different methods.

### 2.5. Erosion Tests

The erosion profile of gel preparation was evaluated with a solution that simulated saliva (solution of KH_2_PO_4_ 0.05 mM at pH 6.75) [26]. The preparation (2 mL) in a transparent vial was weighted and placed in an incubator at 37 °C until gelation occurred. Thereafter, 1 mL aliquot of simulated saliva, pre-equilibrated at 37 °C was added. The test tubes having 1.33 cm^2^ surface area of gel with the solution was placed in the incubator at 37 °C. Every 30 min, the supernatant was completely removed, and the test tube was weighed and another aliquot of the solution was added. The difference in weight of the test tube between any two adjacent time points yielded the change in weight of the gel formulation. The erosion of gel formulation, expressed as percentage of gel dissolved, was obtained according to Equation (1), where *W_i_* and *W_t_* are the initial weight and the weight at time a specific time, respectively.

(1) $$Erosion\;\%=\frac{W_{i}-W_{t}}{W_{i}}\times 100$$

### 2.6. Statistical Analysis

Principal Component Analysis (PCA) was used to evaluate if the attributes could harness information to allow the differentiation of the observations. The dimension reduction promoted by PCA employed a correlation matrix and the extraction of the first three PCs, thereby leading to two graphical representations, namely: the loadings showcasing the eigenvector placement within the two first PCs, and the 3D scatter of the scores. All statistical analysis was conducted considering a significance higher than 5% (*p* < 0.05) and normalization of continuous variables within the range of 0 to 1 in order to standardize the contribution of each attribute.

### 2.7. Determination of the Viscosity

The apparent viscosity (η) of the P407 formulations was measured using a viscometer (ROTAVISC me-vi Complete, IKA^®^-Werke GmbH & Co. KG, Staufen, Germany) equipped with the reducer VOLS-1. The viscometer was connected to a recirculating bath in order to observe a change in the viscosity as a function of temperature.

These measurements were carried out using a customized water bath properly implemented in order to maintain the temperature of the samples at controlled values. The bath consisted in a thermally insulated poly(methyl-methacrylate) tank of internal dimensions of 30 cm × 20 cm × 18 cm (walls thickness of 3.0 mm), equipped with five-sided shells made of extruded polystyrene panels (thickness of 20 mm and thermal conductivity of 0.032 W/m × K). A slab of the same material was used as a cover of the tank. In this configuration, a temperature variation lower than 0.5 °C was observed in approximately 40 min in case of thermal gradient inside/outside the phantom of 22 °C (i.e., temperature of the internal water of 40 °C and ambient air temperature of 18 °C). A 1000 W power thermo-circulator was used to set and control the temperature of water inside the tank (sensitivity 0.1 °C). Additionally, the water temperature was monitored in different positions using digital thermometers with a sensitivity of 0.1 °C.

The heating rate was about 1 °C/min, the viscosity was recorded at regular steps and the speed was set at 40 rpm. The gelling point was determined from the viscosity vs. temperature curve. The linear portion of the curve at high temperature was extrapolated to the temperature axis. The viscosity and shear stress of the formulations were measured at various shear rates at 35 °C. The temperature was maintained within ±0.1 °C. The 2.1 mL of formulations were equilibrated in the cup for 5 min to reach the running temperature prior to each measurement.

### 2.8. Atomic Force Microscopy (AFM) Imaging

AFM imaging was performed in air using a Nanoscope Multimode IIId system (Bruker, Santa Barbara, CA, USA) operating in tapping-mode. 0.5 mL of gel formulations (F10–14) were incubated with 0.1 mL of 8% *w/v* porcine stomach mucin (Sigma-Aldrich) aqueous solution for 10 min at room temperature. Next, 5 μL of the mixture were floated on a freshly cleaved mica disk for 5 min and let dry in a dust free environment. Samples were then visualized at room temperature. The acquired images of the mixtures were compared to the images of gel formulations without mucin and of the mucin aqueous solution alone. AFM images were collected using the RMS amplitude of the cantilever as the feedback signal for the vertical sample position. The RMS free amplitude of the cantilever was approximately 15 nm and the relative set-point above 95% of the free amplitude. Rectangular silicon probes with nominal spring constant around 2.5 N/m (NT-MDT, Moscow, Russia) and cantilever length of 120 μm were used. The cantilever resonance frequency was about 130 kHz. Images were recorded at ~1 Hz line rate and a resolution of 512 × 512 pixels per image was chosen. AFM images were subject to a line-by-line subtraction of linear background to eliminate sample tilt from the images and correct for step-wise changes between individual scan lines by using the Nano-Scope III software (version S.31R1, Bruker, Billerica, MA, USA).

### 2.9. In Vitro Release Studies

Gel formulation (3 mL) was insert in a semipermeable dialysis sack (MWCO 12 kDa). Both side of the membrane were tied up to avoid linkage. The membrane was placed in a graduated cylinder with 75 mL of PBS 1× pre-equilibrated at 37 °C positioned in an incubator at 37 °C under constant stirring. Each sampling time, 0.5 mL of the medium was removed and replaced. Optical absorbance measurements of the medium were carried out with an UV–VIS spectrophotometer (Cary 100 UV–Vis, Agilent Technologies, Santa Clara, CA, USA) in the wavelength interval of 200–340 nm for CHX and 250–450 nm for DOX with steps of 1 nm, using the corresponding calibration curve reported in the SI (Figure S1, see Supplementary Materials). Optical absorbance spectra were acquired using one cuvette filled with PBS 1× as a reference.

## 3. Results and Discussion

### 3.1. Gel Preparation, Determination of T_sol/gel_ and pH

In order to develop the formulation of a thermosensitive drug delivery system based on P407, we firstly, investigated the influence of the concentration of the P407 alone and in presence of additives on the transition temperature. Table 1 shows the sol-gel transition temperature (T_sol-gel_) of different formulations with various additives prepared in ultrapure water. The reported T_sol/gel_ were obtained as average between the results obtained from the two different methods A and B (Table S1, see Supplementary Materials). It is important to underline that, in most of cases, the difference between the two methods were approximately about 2 °C, in few cases about 1 °C. In addition, method B reported in all cases the higher temperature.

**Figure 1:**
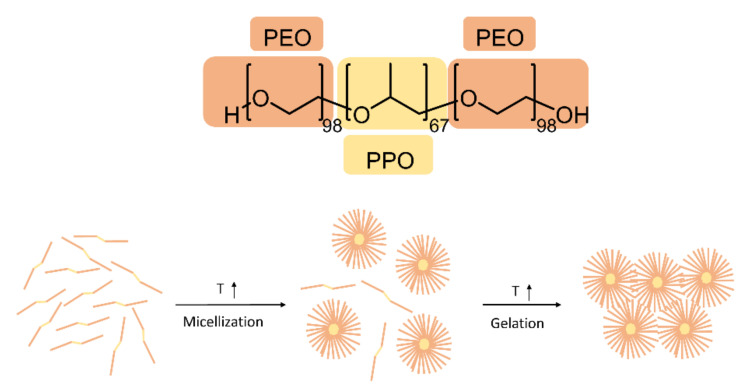
General structure of P407 and representation of micelles formation.

Firstly, we reported the T_sol-gel_ of the gel with decrease percentage of P407 (F1–4). The results showed that increasing the amount of polymer, T_sol-gel_ decreased. In fact, the gelation of the solution occurs via the formation of spherical micelles above a critical temperature. At lower temperature, the P407 is present as monomers in solution. Increasing the temperature, monomers aggregate in micelles through an endothermic process, because of the desolvation of hydrophobic PPO blocks (Figure 1) [27,28].

Our results showed that for F1 (20% of P407) T_sol-gel_ was reached at 21.9 °C, while for F2 (18% of P407) was observed at 25.5 °C. Further decrease of the P407 amount (F3 and F4) induced a T_sol-gel_ higher than 30 °C. Thus, for our purposes, we chose F2 as base for further modification. In fact, despite the great thermogelling properties of P407, it has significant drawbacks, including low gel strength, weak mucoadhesiveness, and rapid dissolution of the gels formed, which limit its performance [29]. To improve the gel properties, hydrophilic polymers were added. In particular, three polysaccharides (XG, E407 and HPMC) and the biocompatible synthetic polymer PVP were selected (F5–9). All of them are FDA approved and are able to influence the micellization process and, consequently, the T_sol-gel_ and the rheological features [29]. Their concentrations were chosen according to the amount described in the literature for pharmaceutical applications [30,31,32,33]. The results showed that the polymers that most change the T_sol-gel_ were XG and HPMC. In fact, 0.2% of XG (F5) decreased the gelation temperature from 25.5 °C to 22.0 °C, while 1% of HPMC (F7) changed to 22.5 °C. E407, instead, did not influenced significantly the T_sol-gel_ of the gel (F6), that remained at 25.0 °C. On the contrary, 4% of PVP caused the lack of gelation in the range of 4–50 °C (F8). This behavior is a consequence of the chemical structure of PVP which is characterized by planar and highly polar side groups due to the amide bond in the lactam ring. Accordingly, when PVP is blended with P407, hydrogen bonds occur between the carbonyl groups of PVP and the hydroxyl groups of P407, disturbing the micellization process. However, when PVP is blended with polysaccharides, PVP/P407 interchain interactions are reduced in favor of interactions with polysaccharides which possess a relatively stronger positive charge than P407, consequently the properties of P407 hydrogels improve [34]. In fact, when 4% PVP was blended in combination with 1% of HPMC (F9), a gel with T_sol-gel_ at 22.0 °C was obtained. The pH of P407 solutions in water was neutral (see Table 1, F1–4). However, the addition of the hydrophilic polymers (F5–9) slightly reduced the pH values, especially in presence of PVP and HPMC which decreased the pH of the solution until 5.74.

The fine control of chemical-physical properties of formulations, such as pH and osmolarity is essential especially in dental medicines, wherein pH value has significant influence on the health of oral cavity tissues [35]. For this reason, in order to prepare gels with physiological pH and osmolarity, water was substituted with PBS 1× (Table 2, F10–14). As reported in literature, the presence of inorganic salts such as Na_2_HPO_4_ and, thus, the increase of the ionic strength causes a reduction in the gelation temperature. This because the anions formed have a great affinity for water, reducing the number of free water molecules in the solutions, and consequently, the solvation of the polymers decreased. Therefore, the interactions between the polymer chains as well as between the polymer aggregates are favored and the gelation process occurs at lower temperatures. In fact, as reported in Table 2, in all cases the transition in PBS occurred at lower temperature than in water. In particular, the preparation with P407 alone (F10) presented a difference of 1.5 °C. The influence of the solvent in the sol-gel transition was particularly evident in presence of HPMC (F13 and F14), because pH alters the structural orientation of HPMC molecules. With increase in pH, HPMC molecules become relatively extended and hence they come into contact with each other even at lower concentration, accelerating the intramolecular interactions [36]. For this reason, in F15 the percentage of HPMC was reduced at 0.5%, in order to maintain the transition temperature above 20.0 °C. On the other hand, the presence of XG (F11) caused only a slight reduction of the gelation temperature (from 22.0 to 21.5 °C), while the formulation with E407 (F12) registered a transition at 23.0 °C, with a decrease of 2.0 °C respect the same formulation in water. Moreover, in all cases, pH resulted comparable to physiological values, in a range between 7.01 and 7.10 (Table 2).

### 3.2. Erosion Behaviors of Gel Formulations

The formulations selected for the erosion test were reported in Table 1 (F2, F6–7, F9) and in Table 2 (F10–14). The erosion tests were performed using a solution of KH_2_PO_4_ 0.05 mM, pH 6.75 that simulate saliva, in order to obtain information regarding the residence time of the gel in the oral cavity. The micellar packing arrangement of P407, indeed, rapidly dissociates in the presence of excess aqueous media leading to the degradation of the gel matrix [37].

**Figure 2:**
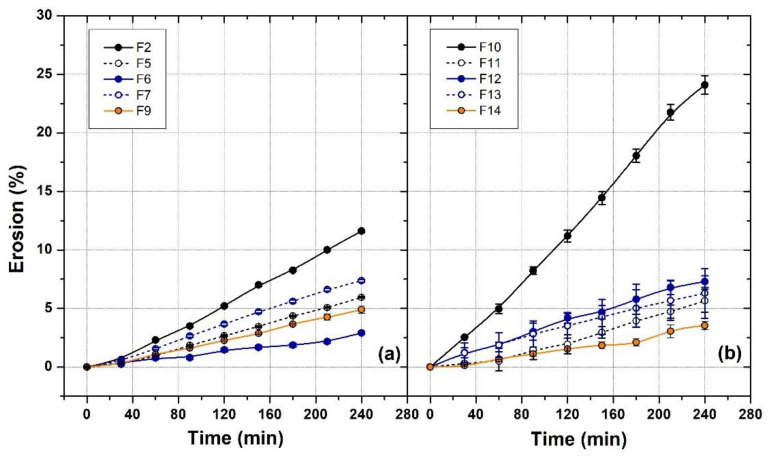
Erosion test with formulation prepared in water (**a**) and in PBS 1× (**b**). The solid lines were trend curves inserted for easy viewing.

Figure 2 reports the results, expressed as percentage weight loss as function of time, of the erosion test for the formulations prepared in water (Figure 2a) or in PBS 1× (Figure 2b). The results showed that in both water and PBS, the faster erosion rate was observed in presence of P407 alone (F2 and F10), while the addition of the hydrophilic polymers reduced the weight loss of about 5% after 4 h. In fact, the added polymers fill the P407 gel network, causing a greater number of cross-links between P407 micelles, and, consequently, increasing the entanglement and rigidity of the gel structure, which requires more time for hydration and dissolution of the polymer chains [29,38]. Moreover, the faster erosion rate was observed in F10, where erosion reached 24% after 4 h, in contrast with F2, where the erosion reached 12%.

### 3.3. Statistical Analysis

**Figure 3:**
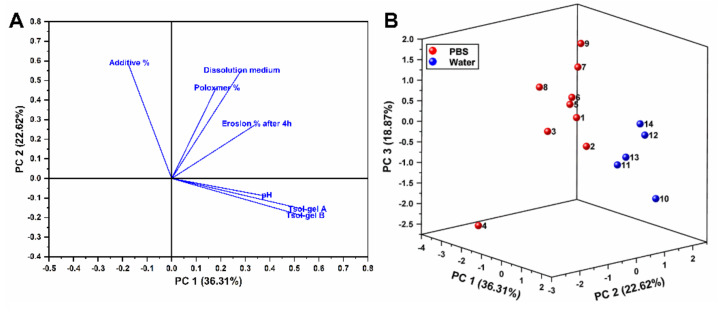
Loading plot of the first two PCs and the eigenvectors of the attributes (**A**). 3D score plot of the first three PCs, being the blue color attributed to the formulations based on water, while the red color of the formulations based on PBS (**B**). The amount of variance explained by each PC is therein represented at the graph label.

Owing to the number of attributes herein investigated, a multivariate approach based on Principal Component Analysis (PCA) was performed with the aim of reducing the dimensions of the datasets and allowing information to be drawn from them. Therefore, the eigenvectors, which are descriptive of the contribution of each attribute to the amount of variance in the PCA were added in a loading plot, whist the scores of the first three PCs were plotted in a 3D model, being each axis representative of a n-numbered PC. The PCA output is presented in Figure 3, wherein the amount of variance explained by each PC is informed in the labels. Furthermore, the correlation matrix which was used for the model is displayed in Table 3, wherein linear correlations can be numerically identified between the attributes.

The results showcased that the first three PCs accounted for 77.80% of the variance of the dataset, while the first two PCs amassed 58.93%. The correlation matrix, on the other hand, showcased that the correlation of the attributes achieved coefficients bellow |0.5|, being the major exception the correlation between T_sol-gel_ A and T_sol-gel_ B, which accounted for a correlation of 0.99711 (Figure 3 and Table 3).

Overall, it could be observed that the eigenvectors of the attributes pH, T_sol-gel_ A and T_sol-gel_ B apparently converged, thereby hinting that the variation of their values could be seemingly proportional. Considering the correlation matrix, it could be seen that the aforementioned attributes correlate to each other with coefficients of pH/T_sol-gel_ A (0.31138); pH/T_sol-gel_ B (0.31187) and T_sol-gel_ A/T_sol-gel_ B (0.99711). Even though the correlation of pH with the other attributes is apparently not adequate, it must be taken into consideration the constrained amount of variance explained by the first two PCs.

Following on, the eigenvectors of P407% and dissolution medium also converged apparently, nevertheless, upon analysis of the correlation matrix, it could be noticed that P407%/dissolution medium correlated with each other with a coefficient of 0.2008. On the other hand, the eigenvectors of the additive% and erosion% after 4 h were not converged with the other eigenvectors. When observing the position of the eigenvectors, it could be hinted that those attributes pH, T_sol-gel_ A and T_sol-gel_ B are better explained by PC1 hence their alignment with the abscissa axis.

Notwithstanding, the eigenvectors of P407% and dissolution medium were seemingly better aligned with the ordinate axis, thence suggesting that PC2 better explains them. In fact, the alignment and magnitude of the eigenvectors is associated with the PC that better associates to them in the explanation of the variance of the dataset. However, considering the restricted amount of variance explained by the first two components, the analysis of the eigenvectors under their dimension reduction could lead to weak correlations. Therefore, the scores were investigated considering the third PC, which explained 18.87% of all variances in the analysis.

The scores of the entries comprising formulations based on water where seemingly adequately separated from those prepared with PBS, and it could be seen that F11, F12, F13 and F14 were closer to each other than to their next-of-kin, i.e., 10. What hints the effect of hydrophilic polymers in the formulation, hence only F11 to F14 had these additives. On the other hand, the scores of F1, F2, F5, and F6 were closer to each other; and somewhat close to F7 and F9. The scores of F3, F4 and F8 were displaced farther apart from the other scores, and were also distant between themselves. Overall, the dataset herein studied suggests that PCA allows the discrimination of the formulations employing water and those based on PBS, however, it only showed hints of discrimination according to the presence of hydrophilic additives in the formulations prepared with water as medium.

### 3.4. Determination of the Viscosity

**Figure 4:**
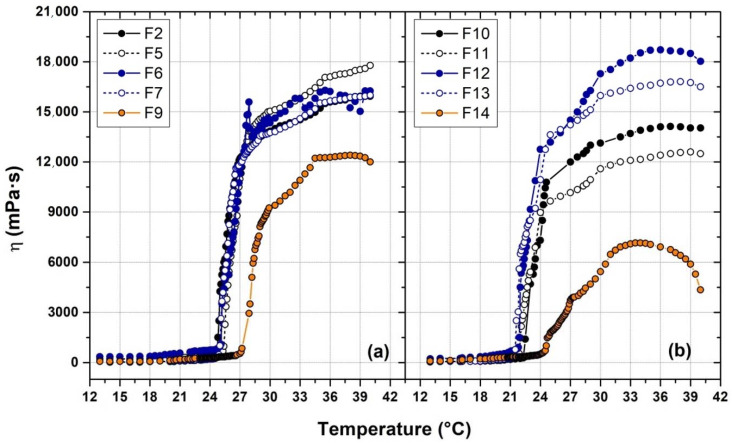
Viscosity profile of formulation prepared in water (**a**) and in PBS 1× (**b**) at different holding temperature. The solid lines are a trend curves inserted for easy viewing.

The viscosity of the formulations F2, F5–7, F9–14 was measured, and the viscosity of these solutions was plotted as a function of the holding temperature (Figure 4). The T_sol-gel_ measured (Table 4) resulted mostly comparable with the one reported previously in Table 1 and Table 2; the slight discrepancy was due to the thermal inertia of the viscosimeter apparatus. Moreover, viscosity evaluation confirmed the effect of PBS in decreasing the T_sol-gel_ respect to water, especially in presence of HPMC (F13) due to the stronger intermolecular interactions previously mentioned. On the other hand, F9 and F14 (both with PVP and HPMC as additives) showed a different T_sol-gel_ values respect to that reported previously (27.0 °C in water and 25.0 °C in PBS against to 22.0 and 19.5 °C, respectively) and the final viscosity of the gel is significantly lower than in the other formulations. This effect could be explained by the PVP characteristic of decreasing the solution viscosity with increasing the temperature [39]. The effect is particularly evident F14, where the polymer molecules are less solvated than in water due to the salting-out effect induced by inorganic salts of PBS and, consequently, the negative influence of PVP on the P407 micellar packaging is higher. Moreover, Figure 4 shows no significant difference in term of viscosity after T_sol-gel_ in water medium exception made for F9, while in PBS the viscosity reached in the formulations tested showed significantly different values.

**Figure 5:**
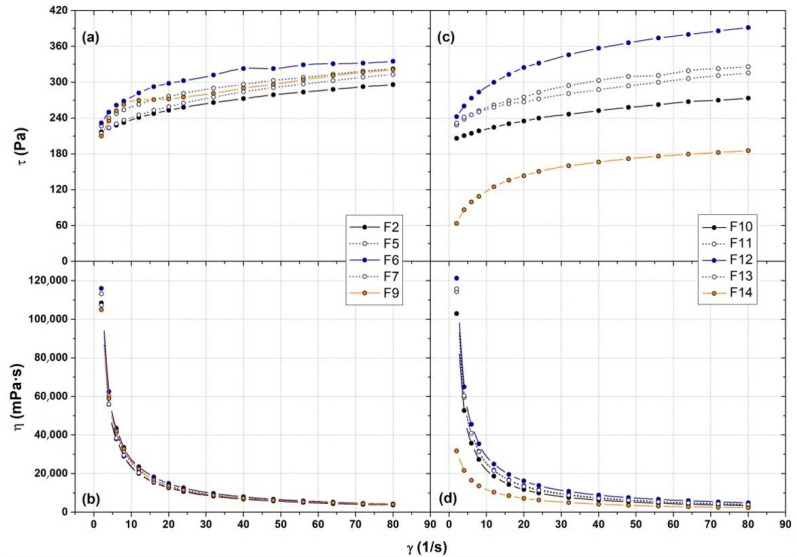
Dynamic viscosity evaluation of the formulations prepared in water (**a**,**b**) and in PBS 1× (**c**,**d**) at different share rate at 35 °C.

In order to investigate better the viscoelastic properties of P407 formulations, another viscosity experiment was conducted, setting the fixed temperature at 35.0 °C and increasing the shear rate γ up to 80 s^−1^. All the formulations at 35.0 °C, both in water and PBS medium, exhibited a non-Newtonian pseudoplastic property, as shown in Figure 5. In particular, all the tested hydrogels possess a shear-thinning flow behavior by which the sample’s viscosity becomes lower at higher γ (Figure 5c,d). This is the most common type of non-Newtonian behavior of fluids and is seen in many industrial and everyday applications [40,41]. Moreover, these formulations are characterized by a yield stress which is a consequence of the high molecular associations and interactions between the polymer chains. The yield stress is defined as the minimum force that must be applied to a sample to start to flow and it is of vital importance for many practical issues and applications, e.g., for quality control of final products (gel stiffness) or for optimizing the production process. In fact, the shear yield identified the lowest shear-stress (τ) value above which the material will behave such as a fluid, and below which the material will act similar to a solid [41,42]. The yield stress is not a material constant but depends on the measuring and analysis method used. According to our results, all the formulations presented yield stress value around 220 Pa, indicating a good stiffness of the gel. The addition of the other polymers slightly increased the yield stress value, especially in PBS medium, confirming the higher toughness respect to the gel with P407 alone. Only formulation F14 showed a sharp decrease of the yield stress (below 70 Pa), confirming the PVP ability of decreasing the solution viscosity according to the temperature.

### 3.5. AFM Imaging

The morphology of different hydrogel formulations (F10–14), alone and in the presence of porcine stomach mucin, was also investigated by Tapping Mode Atomic Force Microscopy (TM-AFM) in order to evaluate the mucoadhesive properties of the polymers [43].

**Figure 6:**
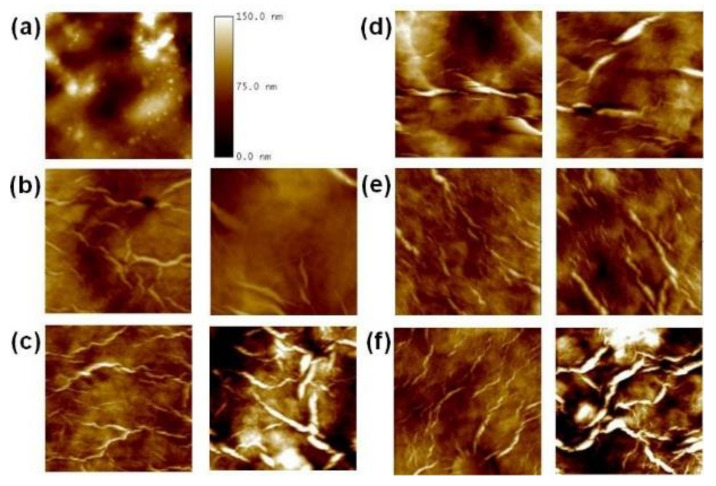
TM-AFM topography images of different hydrogel formulations on a mica support visualized in air. (**a**) Porcine stomach mucin. From (**b**) to (**f**) different hydrogels alone (on the left side) and in the presence of mucin (on the right side). (**b**) F10; (**c**) F11; (**d**) F12; (**e**) F13; (**f**) F14. For all the images: scan area 5 × 5 μm^2^, vertical scale 150 nm.

As an example, in Figure 6a, an AFM topography image of porcine gastric mucin on a freshly cleaved mica support is reported. The sample surface appears quite flattened with aggregated chains in spherical-like structures of different dimensions without the presence of fiber-like structures.

On the contrary, AFM topography images of all the hydrogel formulations (Figure 6, images on the left side) showed a number of well-defined fiber-like structures on their surface similar in conformation and dimensions, independently from the used additives. Fiber-like structures have, on average, length of several microns and thickness in the order of tens of nanometers.

The mixture of hydrogel and porcine gastric mucin (Figure 6, images on the right side) showed still the presence of well-defined fiber-like structures on the surface of all the studied samples even if the fibers size changed in the analyzed formulations and the fiber number increased as well. In particular, as reported in Figure 6c,f, in the presence of XG (F11) and mostly of PVP (F14) as additives, the chains aggregation in fiber-like structures appears more evident: in these samples fibers-like structures increased in size and thickness with respect to the other ones thus suggesting stronger interactions between mucin and the gel network. In the aggregation process, the hydrophobic interactions between these polymers and mucin are supposed to play a key role.

TM-AFM thus confirmed the mucoadhesive properties of XG and the combination of HMPC and PVP, as also already reported in the literature [44,45]. On the other hand, AFM imaging did not show significant interactions between mucin and E407 (F12) or HPMC (F13), thus indicating a poor adhesive capability of these polymers with respect to XG and PVP.

### 3.6. In Vitro Release Studies

**Figure 7:**
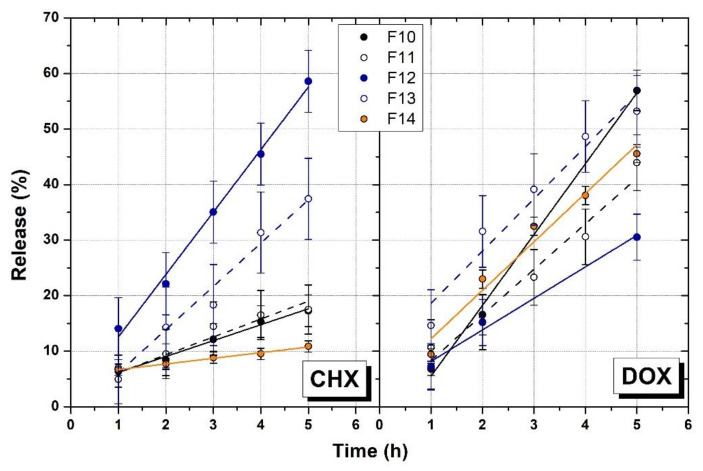
Cumulative percentage of CHX and DOX released over the time. Data are expressed ad mean ±S.D. for *n* = 3.

The percentage of CHX and DOX released in vitro was regressed against time, using UV-vis absorption analysis. Independently from the gel formulations, in-vitro release of CHX and DOX from P407 gel formulations followed first order kinetic [46] at 37 °C, reaching a plateau after 24 h (Figures S2 and S3, see Supplementary Materials). However, in the first 5 h, linearly with time was observed approaching a zero order profile model (Figure 7). Zero order release rate constant (k_0_, μg/h) were calculated for all the formulations and are listed in Table 5. As far as concerned DOX data, it was evident that formulations with additives (F13–14) released this drug at a slower rate. This effect could be attributed to the ability of hydrophilic additives to form intermolecular interactions that affect the molecular orientation of the P407 gel matrix and, consequently, change the arrangement extramicellar aqueous channels by which the hydrophilic molecules are released by their passage through [47]. On the other hand, in the case of CHX, the overall drug release was slower than DOX, and, with exception of PVP (F14), the other additives made this drug release faster than P407 alone (F10). This is particularly evident in presence of E407 (F12) and HPMC alone (F13). These data revealed that the release profile of a drug is strictly correlated with the nature of the loaded molecule and its ability in making interactions with the gel network, as shown in Figure S4 (see Supplementary Materials).

**Figure 8:**
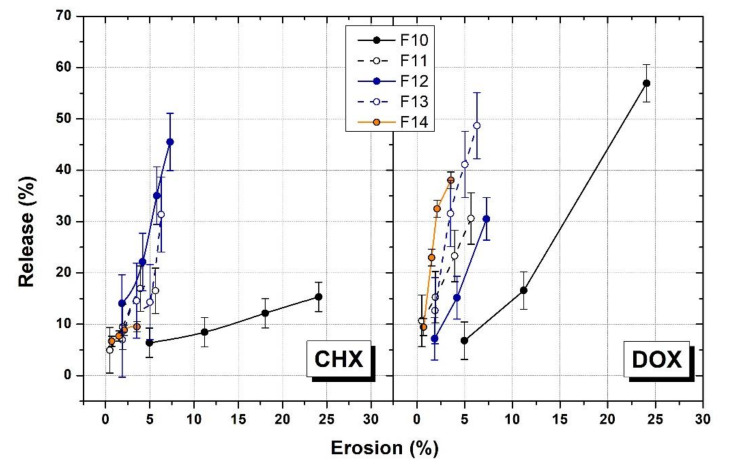
Correlation of cumulative percent gel eroded, and cumulative percent released of CHX and DOX.

In order to understand better the release mechanism from the P407 formulations, the percent of drug release in PBS was plotted as a function of percent of gel eroded at 37 °C in simulated saliva (Figure 8) making the assumption that the release and erosion profiles in PBS and in KH_2_PO_4_ solution are similar.

In both cases for CHX and DOX, the analysis did not show a linear correlation between the rate of drug released and the rate of gel dissolution for all the formulations. However, especially in the case of DOX, the amount of drug released increased as the gel erosion percent increased. This suggests that the gel dissolution plays an important role in the drug release process, but the drug profile release is also strongly influenced by the intermolecular interactions that occur between the polymer network and the drug molecules.

## 4. Conclusions

In conclusion, we reported the physical-chemical characterization of a series of P407-based hydrogel formulated in water and in PBS with the aim of modulating the final pH and to evaluate as the addition of hydrophilic polymeric excipients influence on thermogelling properties. The results showed that T_sol-gel_ is affected both from the dissolution medium and the excipients. In particular, we found that PBS causes a reduction of the gelation temperature due to the presence of inorganic salts that increase the ionic strength of the system. As far as concerned the excipients, HPMC was the polymer that mostly decrease the transition temperature of the formulation especially in PBS, where the extended conformation of HPMC facilitates the intermolecular interactions. On the other hand, PVP alone caused the lack of gel formation. These data were also confirmed by viscoelastic measurements that also highlighted the PVP ability to reduce the viscosity of the gel. Erosion tests showed that all the hydrophilic polymers added are able to reduce the erosion rate probably due to higher rigidity of the gel structure in presence of the excipients. However only XG and PVP impart mucoadhesive properties to P407-based formulation, in fact, TM-AFM images showed significant interactions between these two polymers and mucin. Finally, the influence of the hydrophilic excipients on the release profile of CHX and DOX was evaluated. The results showed that while the addition of excipients decreased the release rate of DOX, as CHX concerned, the release profile is influenced by the intermolecular interactions that occur between the polymer network and the drug molecules.

## Figures and Tables

**Table 1:** T_sol-gel_ and pH of P407 formulations with additives prepared in ultrapure water.

ID Formulation	P407%	Additive%	T_sol/gel_ (°C)	pH
F1	20	-	21.9	7.19
F2	18	-	25.5	7.09
F3	15	-	38.5	7.05
F4	13	-	- *	6.95
F5	18	XG 0.2	22.0	6.45
F6	18	E407 0.2	25.0	6.61
F7	18	HPMC 1	22.5	5.89
F8	18	PVP 4	- *	5.82
F9	18	PVP 4–HMPC 1	22.0	5.74

* The gelation did not occur in the range 4–50 °C.

**Table 2:** T_sol-gel_ and pH of P407 formulations with additives prepared in PBS 1×.

ID Formulation	P407%	Additive%	T_sol/gel_ (°C)	pH
F10	18	-	24.0	7.10
F11	18	XG 0.2	21.5	7.08
F12	18	E407 0.2	23.0	7.01
F13	18	HPMC 0.5	22.0	7.11
F14	18	PVP 4–HMPC 1	19.5	7.01

**Table 3:** Correlation matrix of the attributes used in PCA analysis. A% stands for additive %, DM stands for dissolution medium, and E% after 4 h stands for erosion after 4 h.

	A%	DM	pH	T_sol-gel_ A	T_sol-gel_ B	E% After 4 h	P407%
**A%**	1	0.3146	−0.37182	−0.17998	−0.1979	−0.17595	0.25544
**DM**	0.3146	1	0.4966	0.11061	0.09166	0.45019	0.2008
**pH**	−0.37182	0.4966	1	0.31138	0.31187	0.18601	−0.10465
**T_sol-gel_ A**	−0.17998	0.11061	0.31138	1	0.99711	0.24508	0.22828
**T_sol-gel_ B**	−0.1979	0.09166	0.31187	0.99711	1	0.21857	0.17585
**E% after 4 h**	−0.17595	0.45019	0.18601	0.24508	0.21857	1	0.25089
**P407%**	0.25544	0.2008	−0.10465	0.22828	0.17585	0.25089	1

**Table 4:** T_sol-gel_ range measured by viscometer.

ID Formulation	T_sol/gel_ (°C)
F2	24.7–26.9
F5	25.3–27.8
F6	25.1–27.5
F7	24.8–26.4
F9	27.2–29.8
F10	22.4–24.7
F11	21.9–24.1
F12	21.8–24.0
F13	21.5–24.4
F14	24.5–31.0

**Table 5:** R^2^ and K_0_ constant (μg/h) of different gel formulations.

ID Formulation	Additive%	CHX		DOX	
		R^2^	K_0_ (μg/h)	R^2^	K_0_ (μg/h)
F10	-	0.99	2.86 ± 0.17	0.99	12.75 ± 0.74
F11	XG 0.2	0.97	3.22 ± 0.51	0.96	8.19 ± 0.86
F12	E407 0.2	0.99	11.25 ± 0.47	0.98	5.67 ± 0.57
F13	HPMC 0.5	0.97	7.79 ± 0.73	0.96	9.17 ± 0.80
F14	PVP 4–HMPC 1	0.99	1.05 ± 0.05	0.96	8.73 ± 0.86

## Data Availability

The data presented in this study are available on request from the corresponding author.

## Supplementary Materials

The following supporting information can be downloaded at: https://www.mdpi.com/article/10.3390/polym14173624/s1, Table S1. T_sol-gel_ obtained according to method A and B of P407 formulations with additives prepared in water and PBS; Figure S1: Calibration curve of CHX and DOX in PBS 1×; Figure S2: Cumulative percentage CHX released over the time. Data are expressed ad mean ±S.D. for *n* = 3; Figure S3: Cumulative percentage DOX released over the time. Data are expressed ad mean ±S.D. for *n* = 3; Figure S4. Absorbance spectrum of CHX in the studied formulations (F10–F14).
