# Supplementary material for: Poloxamer-Based Hydrogel as Drug Delivery System: How Polymeric Excipients Influence the Chemical-Physical Properties
Source: Polymers (Basel). 2022 Sep 1;14(17):3624. doi: 10.3390/polym14173624 (PMC9460339; doi:10.3390/polym14173624)
Supplement: Supplementary file 1 [file polymers-14-03624-s001.zip › polymers-1853614-supplementary.pdf]

Supplementary Materials

Poloxamer-Based Hydrogel as Drug Delivery System: How Polymeric Excipients Influence the Chemical-Physical Properties

Elisa Brambilla [†], Silvia Locarno [†,*], Salvatore Gallo, Francesco Orsini, Marco Farronato, Carolina Pini, Douglas Vieira Thomaz, Cristina Lenardi, Marco Piazzoni and Gianluca Tartaglia

Table S1. $T_{sol\text{-}gel}$ obtained according to method A and B of P407 formulations with additives prepared in water and PBS.

ID formulation	P407 %	Additive %	$T_{sol/gel}$ (°C)		
			Method A	Method B	Average
F1	20	-	21.3	22.5	21.9
F2	18	-	24.5	26.6	25.5
F3	15	-	38.5	>40	38.5
F4	13	-	-*	-*	-*
F5	18	XG 0.2	21	23	22.0
F6	18	E407 0.2	24	26	25.0
F7	18	HPMC 1	22	23	22.5
F8	18	PVP 4	-*	-*	-*
F9	18	PVP 4 - HMPC 1	21	23	22.0
F10	18	-	23	25	24.0
F11	18	XG 0.2	21	22	21.5
F12	18	E407 0.2	22	24	23.0
F13	18	HPMC 0.5	21	23	22.0
F14	18	PVP 4 – HMPC 1	19	20	19.5

* The gelation did not occur in the range 4-50 °C.

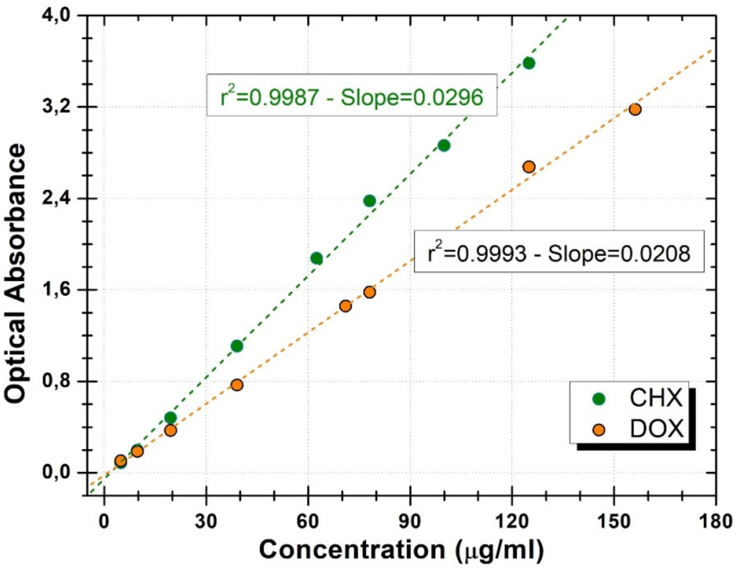

Figure S1. Calibration curve of CHX and DOX in PBS 1x

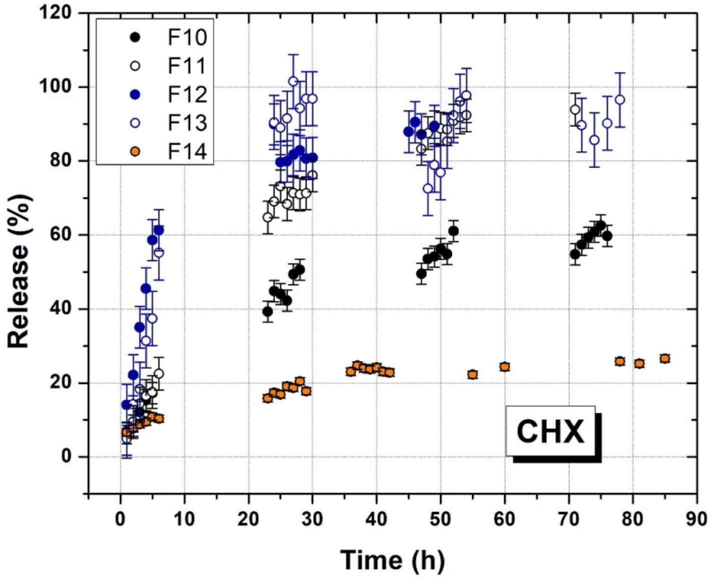

Figure S2. Cumulative percentage CHX released over the time. Data are expressed ad mean ± S.D. for n = 3.

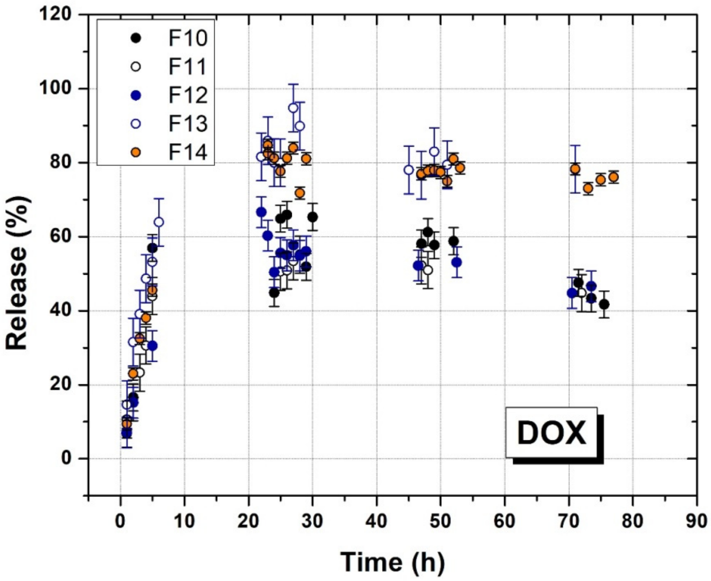

Figure S3. Cumulative percentage DOX released over the time. Data are expressed ad mean ± S.D. for n = 3.

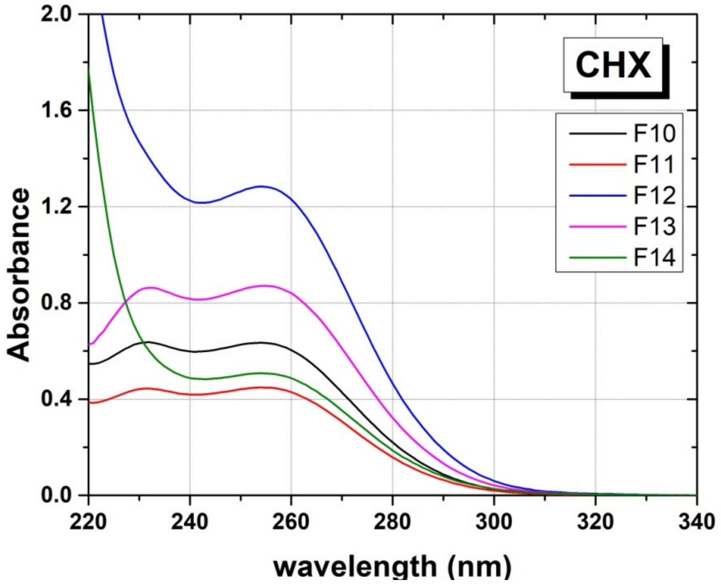

Figure S4. Absorbance spectrum of CHX in the studied formulations (F10-F14).

The analysis highlighted the ability of CHX of interacting with the polymers, in fact, the presence of the polymers changed the absorbed peak of the molecule. In particular this was evident in presence of E407 (F12) and HPMC alone (F13) which could explain the faster release rate observed in the main the main text.
